# Supplementary material for: Pyruvate Dehydrogenase Kinase 1 inhibition mediated oxidative phosphorylation enhancement in cartilage promotes osteoarthritis progression
Source: BMC Musculoskelet Disord. 2023 Jul 20;24:597. doi: 10.1186/s12891-023-06585-6 (PMC10357736; doi:10.1186/s12891-023-06585-6)
Supplement: Supplementary file 1 — Additional file 1. [file 12891_2023_6585_MOESM1_ESM.zip › MF.Gsea.1653627316242/GOMF_ISOPEPTIDASE_ACTIVITY.html]

Details for gene set GOMF\_ISOPEPTIDASE\_ACTIVITY[GSEA]

|  || Dataset | 55457\_collapsed\_to\_symbols.cls.cls #OA\_versus\_normal.cls.cls #OA\_versus\_normal\_repos |
| Phenotype | cls.cls#OA\_versus\_normal\_repos |
| Upregulated in class | OA |
| GeneSet | GOMF\_ISOPEPTIDASE\_ACTIVITY |
| Enrichment Score (ES) | 0.40902743 |
| Normalized Enrichment Score (NES) | 1.2567229 |
| Nominal p-value | 0.19058296 |
| FDR q-value | 0.4605526 |
| FWER p-Value | 1.0 |
Table: GSEA Results Summary

  

Fig 1: Enrichment plot: GOMF\_ISOPEPTIDASE\_ACTIVITY      
 Profile of the Running ES Score & Positions of GeneSet Members on the Rank Ordered List

  

| SYMBOL | TITLE | RANK IN GENE LIST | RANK METRIC SCORE | RUNNING ES | CORE ENRICHMENT || 1 | COPS6 | COP9 signalosome subunit 6 [Source:HGNC Symbol;Acc:HGNC:21749] | 122 | 0.885 | 0.1674 | Yes |
| 2 | HINT1 | histidine triad nucleotide binding protein 1 [Source:HGNC Symbol;Acc:HGNC:4912] | 369 | 0.718 | 0.2918 | Yes |
| 3 | STAMBP | STAM binding protein [Source:HGNC Symbol;Acc:HGNC:16950] | 1227 | 0.471 | 0.3194 | Yes |
| 4 | BRCC3 | BRCA1/BRCA2-containing complex subunit 3 [Source:HGNC Symbol;Acc:HGNC:24185] | 1438 | 0.437 | 0.3905 | Yes |
| 5 | EIF3H | eukaryotic translation initiation factor 3 subunit H [Source:HGNC Symbol;Acc:HGNC:3273] | 2911 | 0.259 | 0.3281 | Yes |
| 6 | SENP2 | SUMO specific peptidase 2 [Source:HGNC Symbol;Acc:HGNC:23116] | 3040 | 0.246 | 0.3673 | Yes |
| 7 | PSMD14 | "proteasome 26S subunit, non-ATPase 14 [Source:HGNC Symbol;Acc:HGNC:16889]" | 3117 | 0.238 | 0.4090 | Yes |
| 8 | COPS5 | COP9 signalosome subunit 5 [Source:HGNC Symbol;Acc:HGNC:2240] | 4261 | 0.131 | 0.3466 | No |
| 9 | GGT1 | gamma-glutamyltransferase 1 [Source:HGNC Symbol;Acc:HGNC:4250] | 5177 | 0.058 | 0.2871 | No |
| 10 | PRPF8 | pre-mRNA processing factor 8 [Source:HGNC Symbol;Acc:HGNC:17340] | 5287 | 0.049 | 0.2884 | No |
| 11 | EIF3F | eukaryotic translation initiation factor 3 subunit F [Source:HGNC Symbol;Acc:HGNC:3275] | 5950 | -0.001 | 0.2373 | No |
| 12 | PSMD7 | "proteasome 26S subunit, non-ATPase 7 [Source:HGNC Symbol;Acc:HGNC:9565]" | 7893 | -0.147 | 0.1160 | No |
| 13 | GGT5 | gamma-glutamyltransferase 5 [Source:HGNC Symbol;Acc:HGNC:4260] | 9361 | -0.261 | 0.0542 | No |
| 14 | USPL1 | ubiquitin specific peptidase like 1 [Source:HGNC Symbol;Acc:HGNC:20294] | 9694 | -0.291 | 0.0866 | No |
| 15 | SENP3 | SUMO specific peptidase 3 [Source:HGNC Symbol;Acc:HGNC:17862] | 9700 | -0.292 | 0.1445 | No |
| 16 | DESI1 | desumoylating isopeptidase 1 [Source:HGNC Symbol;Acc:HGNC:24577] | 11617 | -0.519 | 0.0996 | No |
Table: GSEA details [plain text format]

  

Fig 2: GOMF\_ISOPEPTIDASE\_ACTIVITY      
 Blue-Pink O' Gram in the Space of the Analyzed GeneSet

  

Fig 3: GOMF\_ISOPEPTIDASE\_ACTIVITY: Random ES distribution      
 Gene set null distribution of ES for **GOMF\_ISOPEPTIDASE\_ACTIVITY**
